# In this month’s Bulletin

**DOI:** 10.2471/BLT.22.000322

**Published:** 2022-03-01

In the editorial section, Benjamin Mason Meier et al. (178) argue for a closer link between evidence and global health laws affecting travel in the context of circulating variants of concern. Ren Minghui and Feng Zhao (179) point out that mental health could be better prioritized in humanitarian, peacebuilding and development programmes. Samira Choudhury et al. (180) advocate for substantive research on health impacts of chemical contaminants in food.

In the news section, Andréia Azevedo Soares (183–184) reports on rights-based health service provision for migrants in Europe. Tina Musuya talks to Tatum Anderson (185–186) about her work in community-led reforms to prevent violence against women.

## Albania, Armenia, Benin, Burundi, Ethiopia, Haiti, India, Jordan, Malawi, Nepal, Senegal, Timor-Leste, Togo, Uganda, United Republic of Tanzania

### Anaemia in women of reproductive age

Md Mehedi Hasan et al. (196–204) assess progress towards global nutrition targets.

## Bangladesh, India, Nepal, Pakistan, Sri Lanka

### Chronic obstructive pulmonary disease and chronic bronchitis 

Prashant Jarhyan et al. (216–230) conduct a systematic review to establish prevalence estimates.

## Kazakhstan, Kyrgyzstan, Tajikistan, Ukraine 

### Naloxone use at witnessed overdoses

Paul Dietze et al. (187–195) report outcomes of a cohort study.

## Paraguay 

### Underreporting of congenital syphilis

Katherine Heath et al. (231–236) produce new estimates to inform surveillance and service provision.

## Zambia 

### Active case-finding for tuberculosis 

Patrick S Lungu et al. (205–215) track continuity of services during the COVID-19 pandemic.

